# Connecting the dots: relationship between heart rate and overall dynamic body acceleration in free-ranging cattle

**DOI:** 10.1093/conphys/coae085

**Published:** 2024-12-19

**Authors:** L J Niccolai, Olivier Devineau, Alexandra Thiel, Barbara Zimmermann, L Alina Evans

**Affiliations:** Department of Forestry and Wildlife Management, University of Inland Norway, Evenstad, NO-2480 Koppang, Norway; Department of Forestry and Wildlife Management, University of Inland Norway, Evenstad, NO-2480 Koppang, Norway; Department of Forestry and Wildlife Management, University of Inland Norway, Evenstad, NO-2480 Koppang, Norway; Department of Forestry and Wildlife Management, University of Inland Norway, Evenstad, NO-2480 Koppang, Norway; Department of Forestry and Wildlife Management, University of Inland Norway, Evenstad, NO-2480 Koppang, Norway

**Keywords:** Accelerometry, bio-loggers, Bos taurus, Norway, ruminant

## Abstract

Monitoring physiological indicators including heart rate (HR) is crucial for managing animal welfare across diverse settings, from precision livestock farming to wildlife conservation. HR is a reliable indicator of energy expenditure and stress, yet the invasive nature of HR loggers limits their application in wild and free-ranging species. This study explores whether overall dynamic body acceleration (ODBA), measured with an external accelerometer, can serve as a less invasive proxy for HR. Using free-ranging cattle as a model species in Norway, we examined the relationship between ODBA and HR to assess how external accelerometry might indirectly reflect physiological states in settings that resemble wild conditions. Cattle provide an ideal model because they share some characteristics with wild herbivores, including exposure to diverse terrain and potential predation, whilst offering advantages for handling and sensor retrieval. Our findings showed that low ODBA values corresponded to static behaviours (e.g. standing, ruminating), where small movements caused HR spikes, whilst higher ODBA reflected dynamic activities (e.g. walking, foraging), with HR plateauing. This relationship suggests that ODBA can be used to approximate HR in environments where direct HR measurement is challenging. By using accelerometry to infer HR changes in free-ranging cattle, this study offers insights that could extend to wild species, offering a tool for conservationists to monitor and manage animal health and well-being less invasively.

## Introduction

Understanding animal physiology and energy expenditure is important for effecting conservation of wildlife species. Heart rate (HR) can serve as a reliable indicator of energy use and stress responses (Lewis G. Halsey *et al.,* 2009a; Miwa *et al.,* 2015, 2017). However, accurately measuring HR in wildlife poses significant challenges, primarily due to the invasive nature of traditional HR logging devices (as per the medical definition in Cousins *et al.,* 2019). To address this issue, free-ranging livestock can serve as a model species. These animals face similar environmental pressures as wildlife—exposure to diverse terrain or weather conditions and potential predation—whilst allowing more accessible handling and sensor retrieval. These models provide valuable insights into animal welfare and physiology in extensive environments without disrupting the animals’ natural behaviours, thereby providing an alternative to advance both livestock and conservation research.

Precision livestock farming technologies are advancing these efforts, offering less or non-invasive methods (Bewley *et al.,* 2015; Aquilani *et al.,* 2022; Tzanidakis *et al.,* 2023), combining sensors and data analytics to provide real-time insights into livestock behaviour and physiology (Aquilani *et al.,* 2022). These tools are particularly valuable in extensive systems where animals are typically raised over large, rugged areas with limited human intervention, and they contribute to sustainable ecosystem management where livestock shares habitat with wildlife (Turner *et al.,* 2023).

These systems are generally considered to be more sustainable and welfare-friendly due to their alignment with the Five Freedoms of animal welfare—particularly the freedom to express normal behaviour (Casasús *et al.,* 2011; Herrera *et al.,* 2011; Turner *et al.,* 2023). However, limited human oversight complicates monitoring livestock health and behaviour (Turner *et al.,* 2023). Additionally, unlike in controlled farm environments, free-ranging animals face various external pressures, including changing weather conditions, predators and injuries (Hutchings *et al.,* 2000; Silanikove, 2000; Sevi *et al.,* 2009; Nedeva, 2020). Shifts in movement patterns may signal these stressors, serving as early indicators for intervention (Fogsgaard *et al.,* 2012; Högberg *et al.,* 2019; Rivero *et al.,* 2021; Juge *et al.,* 2024). To address these challenges, farmers and researchers have increasingly turned to bio-loggers (e.g. accelerometers, HR loggers) to monitor livestock remotely (Börger *et al.,* 2020). Accelerometers, which measure the acceleration of an animal’s body movements, enable the identification of behavioural signatures through correlation with recorded (or observed) behaviours (Robert *et al.,* 2009; Vázquez Diosdado *et al.,* 2015; Kamminga *et al.,* 2018; Cabezas *et al.,* 2022; Versluijs *et al.,* 2023). Combining accelerometry with physiological data from HR loggers or body temperature sensors supports a more comprehensive welfare monitoring, facilitating data-driven management in extensive systems (Odintsov Vaintrub *et al.,* 2021; Kleen and Guatteo, 2023; Beltran *et al.,* 2024).

Whilst accelerometers, including the metric ODBA (Overall Dynamic Body Acceleration), are widely employed to estimate energy expenditure across various species (Halsey *et al.,* 2009b; Qasem *et al.,* 2012; Miwa *et al.,* 2015, 2017), they fall short in quantifying the metabolic cost associated with physiological processes. Integrating HR loggers can provide additional insights into energy expenditure, as HR is closely linked to metabolic rate and stress responses in animals (Lewis G. Halsey *et al.,* 2009a; Miwa *et al.,* 2015, 2017). The implementation of HR loggers presents practical challenges, primarily due to invasiveness and cost considerations (Wikelski and Cooke, 2006; Chmura *et al.,* 2018; Cousins *et al.,* 2019). Additionally, external factors—such as environmental conditions, stressors, individual temperament and overall health—can influence HR independently of movement, underscoring the importance of careful interpretation when using movement proxies for physiological data (Waiblinger *et al.,* 2004; von Borell *et al.,* 2007; Kovács *et al.,* 2014; Halsey and Bryce, 2021; Wascher, 2021). As such, validation and refinement of algorithms are essential for accurate assessments, particularly when physiological stress might not correspond directly to activity levels. By advancing these techniques, researchers can bridge livestock management with conservation, particularly where livestock and wildlife coexist in shared landscapes (Hutchings *et al.,* 2000; Silanikove, 2000; Sevi *et al.,* 2009).

We studied the relationship between HR and ODBA in cattle released for summer grazing in the boreal production forest in Norway. The unique conditions in Norway create specific challenges and opportunities, making free-ranging grazing of beef suckler cows a traditional and practical model species for utilizing rugged landscapes (Eriksson, 2011). The limited availability of arable land in the country has resulted in prioritizing winter feed production over summer grazing close to farms and sending free-ranging cattle into the forest and mountains during the summer.

This study’s primary objective was to understand the relationship between two key metrics: HR (derived from subcutaneously implanted bio-loggers) and ODBA (derived from accelerometry readings). We further aimed to explore how behavioural classifications derived from accelerometer data align with HR data, assessing whether accelerometry alone could provide meaningful insights into physiological states in free-ranging cattle. By exploring the feasibility of using accelerometry data as a proxy for HR, this study offers potential pathways for non-invasive, large-scale welfare monitoring, particularly in remote and challenging environments, or for wild species that are difficult to capture and handle.

## Materials and Methods

### Study area

We collected accelerometry and HR data during the 2022 summer grazing season in the Innlandet county of Norway. This region falls within the boreal forest biome, and is characterized by coniferous forests, mires and lakes, with ~4% of the land base dedicated to agricultural fields. During the summer months, numerous livestock breeders choose to release their suckler cows with calves into the forest, with the aim to maximize the utilization of outfield grazing resources whilst conserving fields near the farm for winter forage production.

**Figure 1:**
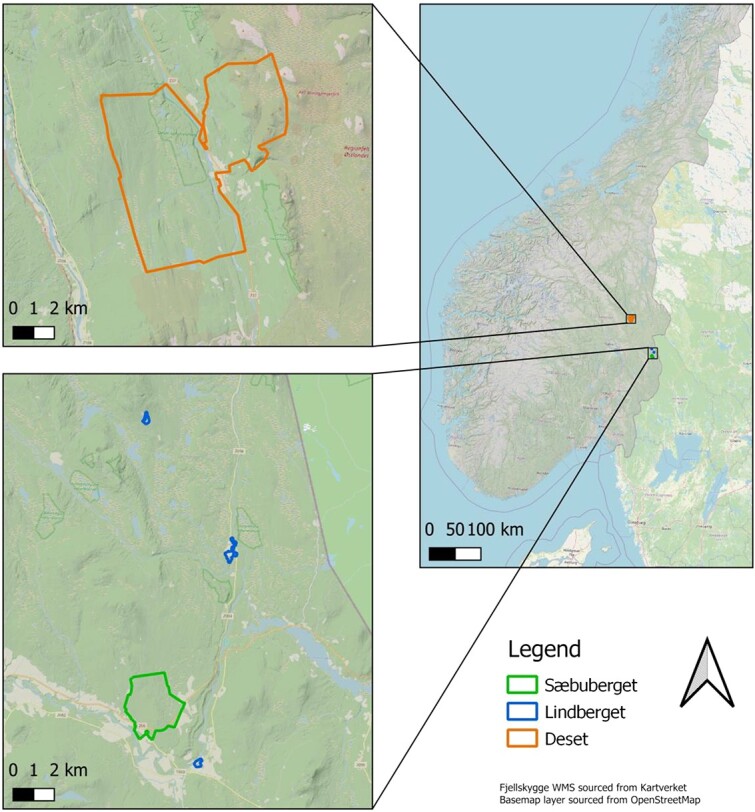
Map of the study area

The study areas, including Deset (~16.4 km^2^), Sæbuberget (~4.7 km^2^) and Lindberget (~6.0 km^2^) (Fig. 1) comprise forest patches of various age classes resulting from clearcutting, soil scarification and other silvicultural methods to boost timber production. The terrain, ranging from 300 to 640 m above sea level, is rugged and interconnected by a network of forest roads (for more details on tree species and habitat, please refer to the ‘Study area details’ in Versluijs *et al.,* 2023). However, the diverse landscapes shaped by these practises pose challenges for free-ranging livestock, as they must navigate uneven terrain punctuated by obstacles such as fallen trees and logging residuals.

### Study animals

All animals in this study were suckler cows from three distinct farms. These cows, accompanied by their calves, were released into summer grazing areas in mid-May 2022, with 23 cows in Deset, 47 in Sæbuberget and 7 in Lindberget.

In May 2022, all adult cows were equipped with virtual fence collars (Nofence®, 2022) and trained to learn virtual fencing following instructions given by the company (Nofence learning process and training pasture, 2018). NoFence collars triangulate the positions of animals (position logged every 15 min if stationary, defined as unchanged across two recordings to conserve power, and every 5 min if moving) through the GNSS positioning system (GPS, GLONASS, GALILEO) and allow the study of movement activity with a motion sensor that yields fine-scale tri-axial accelerometry data (10 Hz). Users have the flexibility to manually define grazing areas with the Nofence app. After training, an acoustic signal is emitted from the collar if individuals approach the virtual fence, followed by a small electric shock (1.0-s duration and 0.2 J or 0.02 V), greatly inferior to that of an electrical fence shock (~3.5 kV; (Verdon *et al.,* 2021)). The batteries lasted for the whole study period and were continuously recharged through solar panels.

These collars weighed 1446 g, which represents 0.3–0.5% of a cow’s body weight and falls far below the recommended threshold of 3–5% for mammals (Soulsbury *et al.,* 2020). Hence, we assumed the devices did not affect the cattle’s behaviour.

Our study focused on 14 of the 77 cows, with 4–5 cows representing each farm. The sample included the following breeds: Hereford (*n* = 5), Jersey (*n* = 1), Norwegian Red (NRF—Norsk Rødtfe, (*n* = 4)) and unique mixed breeds comprising Hereford, Charolais, Angus and NRF genetics (*n* = 4).

### Biosensor programming and surgical implantation

All of our study cows were implanted subcutaneously with HR loggers. These bio-loggers (DST centi-HRT, Star Oddi®, Gardabær, Iceland) (46 × 15 mm and 19 g) were programmed to record HR and subcutaneous temperature with the tag-computer interface (COM-BOX) and using the Star-Oddi’s Mercury software (version 6.41—details on HR measurement methods available in Abecia *et al.,* 2021 and Palacios *et al.,* 2021). Data were sampled at a frequency of 150 Hz with measurements taken every 15 min over a period of 3 weeks (referred to as ‘maintenance period’), followed by minute-by-minute readings for 48 h (referred to as ‘intensive period’). The HR loggers also recorded electrocardiograms (ECGs) during the first 12 h of each intensive period (from 6:00 to 18:00). We manually verified and corrected the recorded ECG data using Star Oddi’s HRT Analyser software (version 1.1.0). According to the user manual of the HRT Analyser programme ((User Manual: HRT Analyser - Analytical software, 2023), this involves visually inspecting the raw ECG data, where peaks are annotated by the bio-logger. The software allows these annotations to be verified and edited by the user, particularly for lower quality ECG data where the bio-logger may have difficulty accurately annotating peaks. This process ensures the data used for analysis is accurate and reliable, and we subsequently only used data from the intensive periods, for which we had corrected HR data.

All implants were sterilized with ethylene oxide gas (Anaprolene AN74i 80 L, Andersen Europe, Kortrijk, Belgium) prior to implantation. Animals were immobilized using either a handling cage or a cattle headlock. Cows were sedated via injection of 10–70 g Xylazine (Rompun vet. Elanco Denmark ApS), and the implantation site was shaved and prepared for aseptic surgical device implantation. Local anaesthesia and analgesia were administered via injection of 300–400 mg Procaine (Procamidor®, Salfarm Scandinavia AS), with 160–360 mg of Meloxicam (Metacam®, Boehringer Ingelheim Vetmedica GmBH, Germany) provided for longer term pain management. A 2-cm incision was made in the skin on the left thorax caudal to the level of the heart, and then surgical haemostats were used to create a subcutaneous pocket for the sensor, which was then inserted. Following implantation, the incision was closed with absorbable monofilament sutures (0 PDS).

The bio-loggers were retrieved following a similar procedure in September of the same year, at the end of the grazing season, and the data were downloaded using a communication box and the Mercury software v6.41 (Star Oddi, Gardabaer, Iceland). Data was time stamped in UTC time zone.

### Accelerometry programming

To get access to the raw accelerometer data, Nofence provided a code that we could use to ‘activate’ the collars to collect and send all data continuously. Due to battery constraints, we used this activation only during bouts of 48 h, at the same time as the intensive periods of the HR measurements. The accelerometer data was time stamped in UTC time zone.

### ODBA pre-processing and ODBA/HR matching

We used the Star Oddi HRT Analyser software to correct logger calculation errors in the ECGs. We then used the *boxfilter* package in R (Ruf *et al.,* 2024) to eliminate remaining outliers and to focus exclusively on high-quality data with quality index (QI) ratings of 0 and 1 (Ruf *et al.,* 2024) and within the expected range for cattle (40–180 beats per minute (Bulitta *et al.,* 2020)). The lowest and highest HR measured from each animal over the 12-h ECG periods are presented in Supplementary Table 2.

Moreover, accelerometry data collected through the Nofence collars underwent pre-processing, following methodologies established in Versluijs *et al.* (2023). Feature calculations and behaviour predictions were derived using algorithms outlined in the same paper (Versluijs *et al.,* 2023). The ethogram utilized in this study is detailed in the appendix of Versluijs *et al.,* 2023. For the ODBA calculation, we first subtracted the mean acceleration from the raw acceleration data for each axis to obtain the dynamic acceleration (dx, dy, dz). Then, the sum of the absolute values of the dynamic acceleration components was used to compute ODBA, as shown in the following formula:


$$ ODBA=\left| dx\right|+\left| dy\right|+\left| dz\right| $$

Here, 𝑥 represents the front-back axis, $ y $ the side-to-side axis and 𝑧 the up-down axis, based on our tri-axial accelerometry data (Versluijs *et al.,* 2023, Fig. 1.).

ODBA was chosen as the measure of choice due to its documented performance in aligning accelerometry and HR data in cattle (Miwa *et al.,* 2015). Based on the literature, ODBA values in movement ecology seldom exceed 3 (g) (Halsey *et al.,* 2009b; Gómez Laich *et al.,* 2011; Meese and Lowe, 2020; Peng *et al.,* 2020; Lauderdale *et al.,* 2021; Bryce *et al.,* 2022). Values above this threshold were deemed biologically incompatible with typical bovine movement patterns (Halsey *et al.,* 2009a; Peng *et al.,* 2020; Wu *et al.,* 2022) and were removed (2.04% of all accelerometer data).

From our complete dataset, which initially contained 7 048 303 raw accelerometry observations at a frequency of 10 Hz and 32 309 raw ECG observations, we processed the data to create a final dataset for analysis. We averaged the accelerometry measurements into 1-min intervals to match the resolution of HR logger. During this process, we discarded HR values that did not have corresponding accelerometry data. This resulted in a final 1-min averaged dataset comprising 12 495 observations.

### Statistical analysis

To account for non-linearity between HR and ODBA and over time, we modelled the relationship between these variables with a generalized additive mixed model (GAM (Kirchner, 2024)) using the *mgcv* package (Wood 2011) in R (R Core Team, 2024—version 4.3.3).

Data visualization indicated that the data approximated a Gaussian distribution with a long right tail, meaning that there were a few high HR values in our dataset. To address this, we employed the scaled t distribution to accommodate heavy-tailed data (via the scat family). Since HR varies over time irrespective of movement, we included a time component where we calculated the elapsed seconds since first measurement per day, by individual (hereby referred to as ‘time index’). We furthermore investigated the potential for a delayed response of HR to ODBA and included l-min lag (lag1) of ODBA and a 5-min lag (lag 5) of ODBA (Supplementary Table 1). We used Akaike Information Criterion (AIC) to compare a list of candidate models (Burnham and Anderson, 2004). Inter-individual variations and repeated measurements were accounted for by integrating a random intercept for each individual cow. We also tested adding breed as a random intercept, but it did not improve model fit and was ultimately excluded. We checked for concurvity and addressed temporal autocorrelation by implementing an AR1 correlation structure (van Rij *et al*. 2022). This AR1 structure was not included in the initial models but was added subsequently to correct for temporal autocorrelation (see Supplementary Table 1).

### Ethical permit statement

Farmers granted written consent for their cows’ participation and collaborated by freely granting access to their Nofence accounts and data.

All procedures were approved by the Norwegian Food Safety Authority (FOTS id 27 543).

## Results

**Figure 2:**
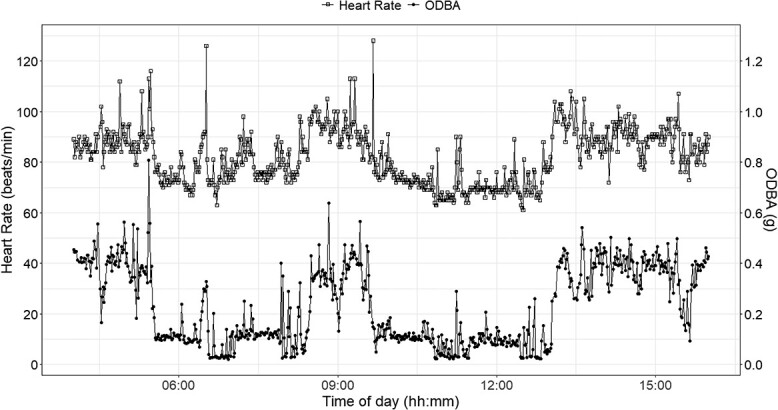
Simultaneous recordings of 1-min HR (open squares) from a subcutaneous Star Oddi logger and 1-min averaged ODBA (closed circles) from a tri-axial accelerometer attached to a Nofence collar in an individual female cow (ID 70489) from June 2022.

In the 1-min average dataset (*n* = 12 495 observations), 80% of HR values (*n* = 9993 values) were rated ‘excellent’ (quality index 0), and 20% (n = 2502 values) were rated ‘good’ (quality index 1). Figure 2 illustrates an example of HR and ODBA fluctuations over a 12-h period for one cow and one ‘intensive’ HR measurement period. The HR ranged between 40 and 173 beats per minute, whilst ODBA ranged between 0.01744 and 0.91838 (g).

We found that the top models included the AR1 structure to account for temporal autocorrelation (Supplementary Table 1). All top AIC models also featured non-linear terms for ODBA and time index. The highest ranked model incorporated smoothed terms for ODBA and time index, their interaction and a random effect for individual differences (Supplementary Table 1). To compare with our top GAM model, we included a linear model to reflect its common use in literature for exploring the HR/ODBA relationship. The linear model, listed as the fourth in Supplementary Table 1, performed worse based on AIC scores (AIC difference of 504.1).

**Figure 3:**
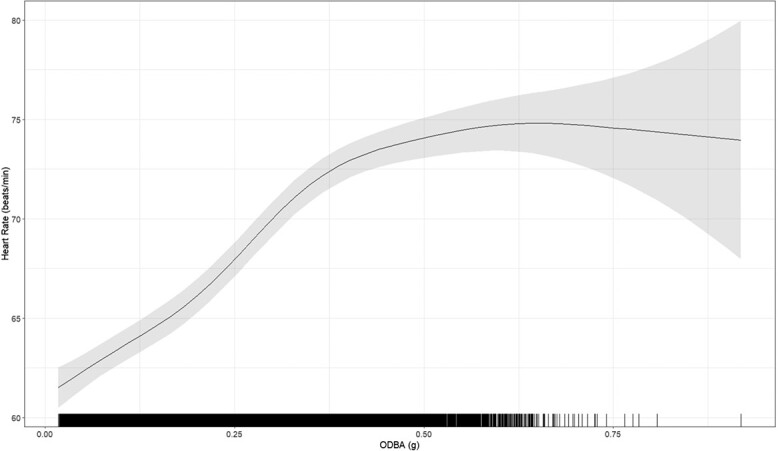
Effect of overall dynamic body acceleration on HR in free-ranging cattle predicted from a GAM. The black solid line represents the predicted mean, and the grey area shows the 95% confidence interval.

Figure 3 illustrates the predicted HR in relation to ODBA from our highest ranked model, with the light grey area representing the 95% confidence interval. The regression is approximately linear for ODBA values <0.4, but then plateaus for higher values of ODBA.

**Figure 4:**
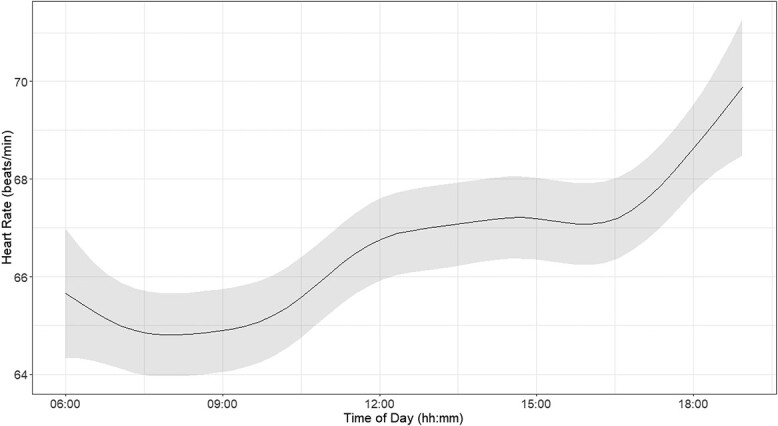
Influence of time of day on predicted HR values of free-ranging cattle. These values are derived from clean data extracted from 12-h ECGs. Grey area shows the 95% confidence interval.

Predicted HR values depending on time index values followed a non-linear trend with low values in the morning and increasing values towards the evening (Fig. 4, see also Supplementary Fig. 2 for average HR differences per individual over the study period).

**Figure 5:**
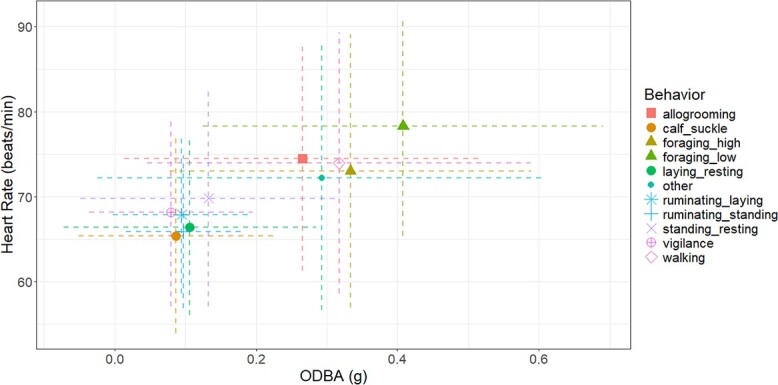
Average predicted HR and ODBA values per behaviour in free-ranging cattle. Dotted lines represent the standard deviation for HR (vertical) and ODBA (horizontal).

Relating HR to behavioural classifications and ODBA based on collar-derived accelerometer data showed that low ODBA and HR values correspond to static behaviours such as calf suckling, ruminating (standing or lying down), standing (resting), lying (resting) and vigilance (Fig. 5). In contrast, higher ODBA and HR values are associated with walking, high and low foraging and other activities (Fig. 5). The ‘Other’ category, which includes running, has the highest ODBA values (Supplementary Fig. 1) due to significant physical exertion and may also include grooming behaviours involving extensive head movement (see Appendix in Versluijs *et al.,* 2023 for a full list of behaviours included in ‘others’). It is important to note that although the highest values recorded fell into this group (Supplementary Fig. 1), the mean ODBA for this group was not the highest (Fig. 5). However, the range of ODBA and HR values associated with the prediction of a given behaviour overlapped substantially (Fig. 5).

## Discussion

In this study, we matched HR data with corresponding tri-axial accelerometer data using GAM models in free-ranging cattle. We observed that the relationship between HR and ODBA was approximately linear for ODBA values up to 0.4, after which it plateaued. Additionally, lower ODBA values were associated with more static behaviours such as calf suckling, ruminating and vigilance, whilst higher ODBA values corresponded to more active behaviours such as foraging (both high and low), walking and other activities including running.

Most studies in the literature present a linear relationship between HR and ODBA (Halsey *et al.,* 2009b; Miwa *et al.,* 2015). Our findings are consistent with this, but we hypothesized more variability in HR at higher ODBA values. It should be noted that our collar placement differs from Miwa *et al.* (2015), and could be attached more loosely, which could possibly contribute to such increased variability across the range of ODBA. Nonetheless, we hypothesized that our results indicate that transitioning from static behaviour to any form of movement requires an initial increase in HR as the body demands more oxygen for muscle activation (Kuhlmann *et al.,* 1985; Evans and Rose, 1988; Laughlin, 1999; Poole *et al.,* 2020). However, once the movement stabilizes, such as in walking at a constant pace, HR tends to plateau (Davies and Harris, 1964; Williamson *et al.,* 1995; Ainslie *et al.,* 2005; Reimoser, 2012). Moreover, the distinction we observed between high and low foraging behaviours in terms of different ODBA is logical. High foraging involves cows consuming leaves from trees, a relatively stationary activity, resulting in lower ODBA values. Low foraging, however, involves walking and repetitive head movements whilst grazing, thus exhibiting higher ODBA values. It would be particularly interesting to investigate transition behaviours, such as getting up, lying down and changing from walking to running, to understand these dynamics better. Like Miwa *et al.* (2015), we found no differences in cattle breeds with similar weights affecting the relationship between HR and ODBA. Interestingly, our HR predictions over time reveal a circadian pattern similar to that observed by Palacios *et al.* (2021) in low-density grazing conditions, which aligns with the free-ranging environment of our cows. It is important to note that in our study, the use of virtual fencing collars did not introduce additional stress related to electric shocks. According to the findings from the Department for Environment, Food and Rural Affairs (2024), the electric shocks from these collars do not have a differential effect on livestock stress. Additionally, Hoag *et al.* (2024) surveyed farmers directly, revealing a consensus that the shock itself is very mild, with more stress observed when collars are initially fitted, particularly in breeds less accustomed to handling, such as beef cattle compared to dairy cattle. In our case, the cows were only exposed to electric shocks during the initial training phase to condition them to the system. Once this training was completed, shocks were not administered regularly. Therefore, the potential impact of the electric shocks on stress levels should be minimal and not affect the study’s measurements of ODBA and HR. This approach ensures that any observed variations in data are not influenced by the virtual fencing system’s operational aspects.

The link between ODBA and HR is crucial because it enables the estimation of energy expenditure with minimal invasiveness, allowing for continuous remote monitoring in free-ranging cattle (Halsey *et al.,* 2009a; Gómez Laich *et al.,* 2011; Qasem *et al.,* 2012; Miwa *et al.,* 2015; Mulvenna *et al.,* 2022). Continuous monitoring of these metrics provides insights into the cattle’s activity levels and physiological states, which are indirect indicators of well-being. For example, deviations in movement patterns or abnormal HR levels can signal stress, illness or discomfort, thereby helping to identify and address welfare issues proactively. Although temperature could be a useful addition, we opted not to include temperature readings in this study, as temperature readings in our study were subcutaneous and not necessarily reflective of true core body temperature, making them less reliable for this purpose. These data are valuable for assessing the health and well-being of free-ranging cattle by providing a comprehensive view of their physiological and behavioural responses in natural environments. Moreover, accurately measuring energy expenditure is challenging when it involves behaviours where the relationship between ODBA and HR is non-linear. As noted by Halsey and Bryce (2021), there is a risk of under or overestimating energy expenditure when it is calculated from uncalibrated proxies such as accelerometry or HR. Our findings suggest a non-linear relationship at higher ODBA values, indicating that current methods may overestimate the energy expenditure of these activities. This potential overestimation needs to be addressed to improve the accuracy of energy expenditure measurements in such contexts. However, it is important to note that cows spent the majority of the time in stationary behaviours (represented by lower ODBA values), where the linear relationship is solid.

Furthermore, we investigated the potential for a delayed HR response to ODBA by including lags in our candidate models. However, neither a 1-min nor a 5-min lag of ODBA improved model fit. Our findings suggest that HR does not show significant delayed responses to ODBA, which may indicate that the HR adaptation to ODBA changes occurs quickly enough to be captured without lags. The 1-min scale might not have been fine enough to model the HR response delay to changes in ODBA, particularly at low ODBA and high HR values. For instance, if a cow runs briefly and then stops, HR should remain elevated and could take up to a few minutes to stabilize, whilst ODBA would return to a low value (Kuhlmann *et al.,* 1985; Bruckmaier and Blum, 1992; Prahesti *et al.,* 2021; Talmón *et al.,* 2023). This discrepancy would lead to an elevated mean HR for low ODBA values, but we did not observe this pattern. Thus, whilst fine-scaled accelerometer data is advantageous for observing detailed behaviour, matching it with coarser data requires compromises. It is crucial to clearly define research questions to select the data resolution that best aligns with the study’s goals.

Our study highlights the importance of continuous monitoring of ODBA and HR in assessing cattle well-being, which is essential for conservation efforts (Alipio and Villena, 2023). By providing insights into energy expenditure and physiological responses, these metrics help in understanding how cattle adapt to their environments, directly impacting their management and the traditional practise of summer free-ranging. Improved data on high ODBA values and less common behaviours, such as running, could enhance our understanding of the full range of cattle responses. The variability in responses we observed, influenced by factors like environmental conditions, psychological stress and individual temperament, or overall age and health, underscores the need for further research. Addressing these factors will strengthen the connection between monitoring data and conservation outcomes. Importantly, free-ranging cattle often share habitats with large carnivores, leading to potential conflicts. By developing algorithms that analyse stress responses in cattle, we can gain detailed insights into stressful situations, such as carnivore encounters, which can inform strategies to mitigate these conflicts. This approach not only contributes to the conservation of the tradition of summer free-ranging cattle but also supports the protection of wildlife. We have a responsibility to care for the animals we put out for free-ranging and improving our monitoring methods will support this goal whilst addressing the challenges posed by large carnivores.

Another important direction for future research is extending the relationship between HR and ODBA to wild species for conservation purposes. Whilst our study focused on free-ranging cattle, the continuous monitoring of these metrics could provide valuable insights into wildlife behaviour and welfare (Kirchner, 2024). Applying these technologies to track the physiological responses of endangered species to environmental stressors, habitat disruptions and human–wildlife interactions could help mitigate conflicts and improve conservation management. Integrating these techniques into wildlife monitoring would enhance models of animal behaviour, supporting more informed conservation decisions and contributing to biodiversity conservation (Beltran *et al.,* 2024). The ability to assess behavioural and physiological patterns from accelerometry data alone has significant applications beyond research. For instance, commercial applications such as Nofence could benefit from this technology, enabling users to monitor and manage cattle welfare. The integration of such technology into everyday farming practises represents a proactive approach to welfare management, promoting the health and productivity of livestock.

In conclusion, our study using GAM models revealed a linear relationship between HR and ODBA up to ODBA values of 0.4, beyond which HR plateaued. Lower ODBA values were associated with static behaviours like calf suckling and ruminating, whilst higher values indicated more active behaviours such as foraging and walking. The discovery of a non-linear relationship between HR and ODBA at high ODBA values provides a more nuanced understanding of how HR and ODBA interact in response to increased physical activity. However, it is noteworthy that cows typically exhibit lower ODBA values, which are better predicted by our models. This finding underscores the robustness of our approach for most of the cows’ behaviour. To further enhance model accuracy and address variability, future studies should include a wider range of high ODBA behaviours. Understanding these high ODBA scenarios is crucial, particularly for studying disturbances and their impact on cattle welfare and productivity.

## Supplementary Material

Web_Material_coae085

## Data Availability

The data and R script to replicate the models of this study are openly available in DataverseNO at https://doi.org/10.18710/8TWPS8.
