# Supplementary material for: Connecting the dots: relationship between heart rate and overall dynamic body acceleration in free-ranging cattle
Source: Conserv Physiol. 2024 Dec 19;12(1):coae085. doi: 10.1093/conphys/coae085 (PMC11655874; doi:10.1093/conphys/coae085)
Supplement: Web_Material_coae085 [file web_material_coae085.pdf]

Connecting the dots: Relationship between heart rate and Overall Dynamic Body Acceleration (ODBA) in free-ranging cattle

Niccolai, Laura J.[1]; Devineau, Olivier[1]; Thiel, Alexandra[1]; Zimmermann, Barbara[1]; Evans, Alina L.[1]

[1] Inland Norway University of Applied Sciences, Department of Forestry and Wildlife Management, Evenstad, NO-2480 Koppang, Norway.

*** Correspondence:**
Laura J. Niccolai

laura.niccolai@inn.no

4 Supplementary materials

Supplementary Table 1: Summary of all the generalised additive mixed and linear models quantifying the effect of Overall Dynamic body Acceleration on free-ranging cattle heart rate. Component column uses the formula notations from the mgcv package. RE refers to the random effect, here the serial ID of each individual.

Components	*AR1 structure*	*AIC*	*AIC_diff*	*AIC_weight*	*df*
HR ~ s(ODBA) + s(time index) +ti(ODBA*time index) + RE	Y	81974.1	0.0	1	40.1
HR ~ s(ODBA) + s(time index) + RE	Y	82253.8	279.2	6.329E-35	30
HR ~ s(ODBA) + RE	Y	82371.8	397.2	9.03E-99	23.2
HR ~ ODBA + time index + ODBA*time index + RE	Y	82478.2	504.1	1	19.9
HR ~ s(lag_ODBA_1) + s(time index) + ti(lag_ODBA_1*time index) + RE	Y	83121.0	1146.4	0	39.5
HR ~ s(lag_ODBA_5) + s(time index) + ti(lag_ODBA_5*time index) + RE	Y	84361.5	2386.9	0	39.5
HR ~ s(ODBA) + RE	N	85492.4	3517.8	0	23.1
HR ~ s(ODBA) + s(index)	Y	87329.3	5354.7	0	13.9

Supplementary Table 2: Summary of the minimum and maximum heart rate values recorded for each individual during the study period using subcutaneous Star Oddi loggers. These values are derived from clean data extracted from 12-hour ECGs. The 'breed' column utilises the following abbreviations: Her (Hereford), Char (Charolais), Ang (Angus), and NRF (Norsk Rødtfe or Norwegian Red).

Serial	*Breed*	*Minimum HR*	*Maximum HR*
70489	Hereford	61	128
71533	Hereford	41	166
73181	Hereford	40	126
73652	Hereford	40	116
74971	Hereford	56	118
74643	NRF	45	142
74719	NRF	40	117
74725	NRF	40	173
84196	NRF	41	136
84288	Jersey	40	128
46883	Her/Char/Ang/NRF mix	40	111
47634	Her/Char/Ang/NRF mix	40	169
47836	Her/Char/Ang/NRF mix	44	116
80017	Her/Char/Ang/NRF mix	42	104

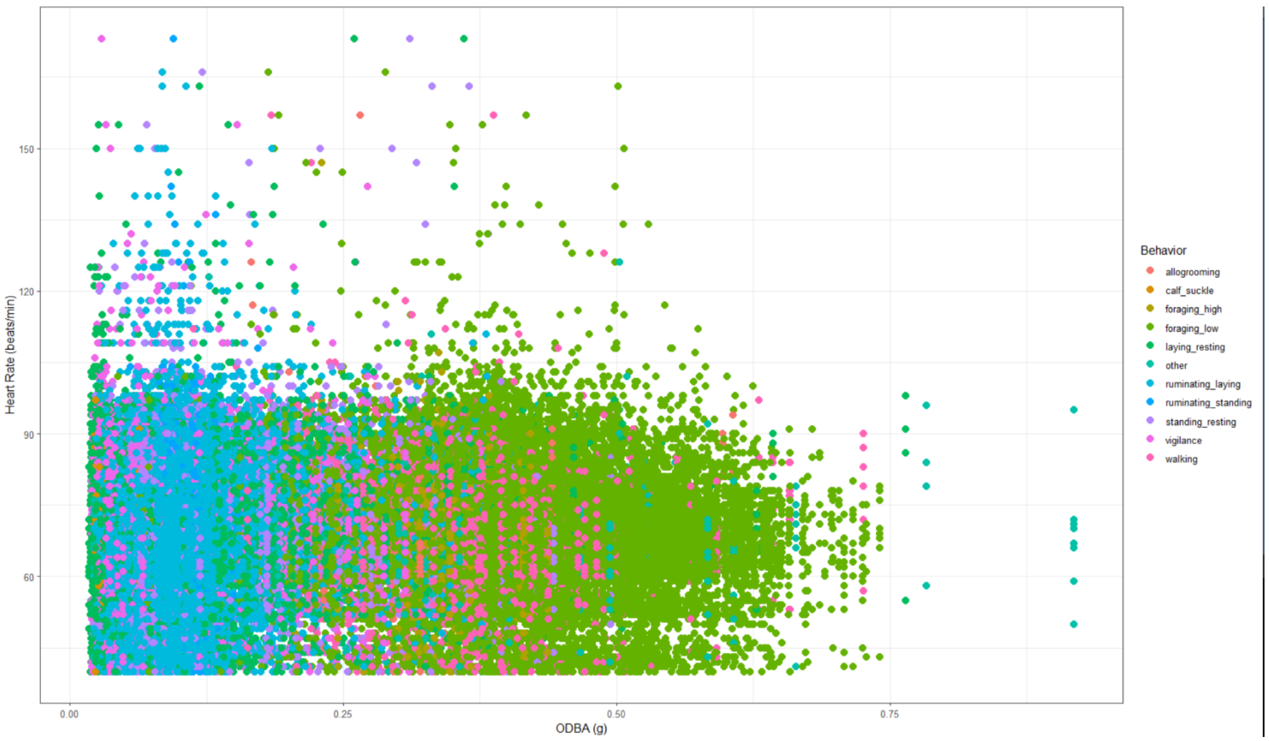

Supplementary Fig. 1: Scatterplot illustrating the raw data from free-ranging cows, with heart rate (HR) values plotted on the y-axis and Overall Dynamic Body Acceleration (ODBA) values on the x-axis. Each data point is color-coded based on the associated behaviour.

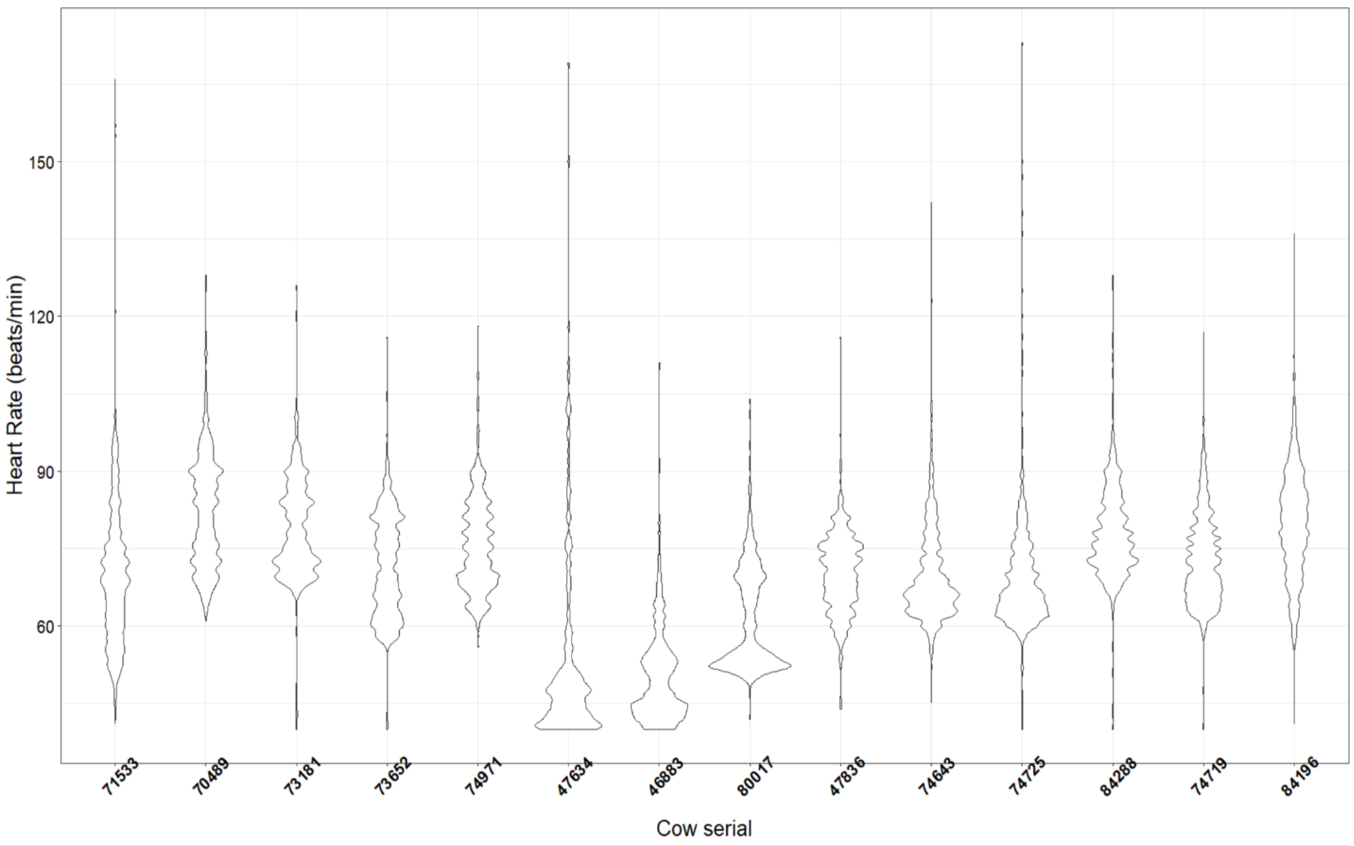

Supplementary Fig. 2: Average heart rate values across the study period (Summer 2022) for each free-ranging cow individual.
